# ULK1 Regulates Melanin Levels in MNT-1 Cells Independently of mTORC1

**DOI:** 10.1371/journal.pone.0075313

**Published:** 2013-09-16

**Authors:** Eyal Kalie, Minoo Razi, Sharon A. Tooze

**Affiliations:** Secretory Pathways Laboratory, London Research Institute, Cancer Research UK, London, United Kingdom; Peking University Health Science Center, China

## Abstract

Melanosomes are lysosome-related organelles that serve as specialized sites of melanin synthesis and storage in melanocytes. The progression of melanosomes through the different stages of their formation requires trafficking of specific proteins and membrane constituents in a sequential manner, which is likely to deploy ubiquitous cellular machinery along with melanocyte-specific proteins. Recent evidence revealed a connection between melanogenesis and the autophagy machinery, suggesting a novel role for members of the latter in melanocytes. Here we focused on ULK1, a key autophagy protein which is negatively regulated by mTORC1, to assess its potential role in melanogenesis in MNT-1 cells. We found that ULK1 depletion causes an increase in melanin levels, suggesting an inhibitory function for this protein in melanogenesis. Furthermore, this increase was accompanied by increased transcription of MITF (microphthalmia-associated transcription factor) and tyrosinase and by elevated protein levels of tyrosinase, the rate-limiting factor in melanin biogenesis. We also provide evidence to show that ULK1 function in this context is independent of the canonical ULK1 autophagy partners, ATG13 and FIP200. Furthermore we show that regulation of melanogenesis by ULK1 is independent of mTORC1 inhibition. Our data thus provide intriguing insights regarding the involvement of the key regulatory autophagy machinery in melanogenesis.

## Introduction

Melanosomes are a type of Lysosome Related Organelle (LRO). As implied from their name, most LROs share some common features with lysosomes, such as an acidic lumenal pH and the presence of lysosomal proteins [1], however LROs are unique to specific specialized cell types. Another important feature often shared between lysosomes and LROs is the origin of their membrane and lumenal content, as many (but not all) LROs are derived from early endosomes, in contrast to other secretory organelles that typically originate from the trans-Golgi network [2]. However, similar to other LROs, melanosomes are also characterized by unique features that clearly distinguish them from lysosomes both functionally and morphologically [3].

Melanosomes progress through four maturation steps. Stage I melanosomes contain intralumenal vesicles and irregular fibrils formed by the melanocyte-specific PMEL protein [4], while at stage II the fibrils are structured into ordered striations along the long axis of the melanosomes [5]. Upon delivery of enzymes such as tyrosinase (TYR) and tyrosinase-related protein 1 (TYRP1), melanin is synthesized and deposited onto the PMEL fibrils, giving rise to thick striations that are characteristic of stage III melanosomes [3,6]. Melanin further accumulates in the organelle until it reaches stage IV, which is a mature (i.e. fully pigmented) melanosome [7]. Melanosome formation utilizes cellular trafficking machinery typically associated with other pathways, in conjunction with specific factors that provide organelle specificity and segregate them from other, more ubiquitous organelles. An example of this comes from the study by Bultema et al, which shows that ubiquitous factors of lysosome biogenesis machinery, i.e. AP-1, AP-3 and BLOC-2, interact with the melanosome-specific proteins Rab32 and Rab38 to specifically drive melanogenesis [8,9].

Recently, a role for proteins associated with autophagy has also been implicated in melanogenesis [10]. Autophagy is a highly conserved degradation process that can be triggered in virtually all cell types in the body under challenging conditions such as nutrient deprivation, hypoxia and accumulation of aberrant protein aggregates [11]. It is a tightly regulated process comprised of several sequential steps, where targeted proteins and organelles are engulfed by double membranes to form vacuoles known as autophagosomes, which subsequently fuse with lysosomes to facilitate the degradation of their content [12]. The different steps in this process are governed by specific autophagy-related (Atg) proteins, which comprise a group of over 35 proteins. In a screen published by Ganesan et al, several Atg proteins have been found to regulate melanin levels in MNT-1 cells [13]. Specifically, depletion of WIPI1, LC3 or Beclin1 from these cells resulted in decreased levels of melanin. A follow-up paper by Ho et al further showed that melanogenesis could be positively regulated by WIPI1 through its inhibitory effect on the mTORC1 complex, which by itself is a negative regulator of melanogenesis [14].

ULK1 is a pivotal player in starvation-induced autophagy, functioning as a link between the nutrient-sensing mTORC1 complex and the initiation of autophagosome formation [15]. ULK1 forms a complex with three additional autophagy proteins: ATG13, FIP200 and ATG101. Under normal conditions, this protein complex is directly bound to, and negatively regulated by, the mTORC1 complex. Upon nutrient deprivation, the ULK complex dissociates from mTORC1, becomes activated and facilitates the induction of autophagosome formation through a mechanism that is still unclear [16]. In light of the recent findings regarding involvement of the autophagy machinery in melanogenesis, and given the known inhibitory role of mTORC1 in this process, we were interested in the possible function of ULK1 in melanogenesis. We show here that depletion of ULK1 in MNT-1 cells results in elevated mRNA levels of MITF and tyrosinase, and increased protein levels of tyrosinase which increases melanin production. Knocking down ATG13 or FIP200 on the other hand does not result in a similar effect, suggesting that ULK1 may inhibit melanogenesis independently of its role in autophagy.

## Materials and Methods

### Cell culture

Human MNT-1 cells were a gift from Prof. Michael Marks, University of Pennsylvania [17]. The cells were cultured in DMEM (Invitrogen) supplemented with 17% fetal bovine serum (Sigma), 10% AIM-V medium (Invitrogen), 0.1mM non-essential amino acid mix (Invitrogen) and 1mM sodium pyruvate (Invitrogen).

### Antibodies and reagents

The following primary antibodies were used for western blotting: Polyclonal anti-ULK1 (Santa Cruz sc-33182), monoclonal anti-TYR (Santa Cruz sc-20035), polyclonal TYRP1 (Santa Cruz sc-25543), polyclonal tubulin (Abcam ab6046), monoclonal Actin (Abcam ab11003), polyclonal anti-FIP200 (Bethyl Labs A301-536A), polyclonal anti-phospho-S6K (Cell Signalling 9250), polyclonal anti-S6K (Cell Signalling 9202), polyclonal anti-phospho-Akt (Cell Signalling 4060),) polyclonal anti-LC3 (Abcam ab48394). Polyclonal anti-ATG13 was generated using the peptide sequence LAVHEKNVREFDAFVETLQ [18]. The secondary antibodies used are Alexa Fluor® 680 Goat Anti-Mouse (Invitrogen, A-21058), HRP-conjugated sheep anti-mouse (GE Healthcare) or HRP-conjugated sheep anti-rabbit (GE Healthcare). Rapamycin was purchased from Millipore (Insolution Rapamycin, 553211), cycloheximide was from Calbiochem.

### siRNA treatments

siRNA knockdown in MNT-1 cells was performed in two rounds of siRNA transfection, on day 1 and day 3 after plating, and analysis of melanin and proteins was carried out at day 6. Transfection was carried out by incubating the cells 4h with a mix of 50nM siRNA and 2.5µl/ml Lipofectamine (Invitrogen, 11668-019) in OptiMEM (Invitrogen, 31985-047). All siRNA duplexes were obtained from Dharmacon, targeting the following proteins: TYR (siRNA pool containing J-012555-05, J-012555-06, J-012555-07, J-012555-08), ULK1 (D-005049-04 or D-005049-08), ATG13 (J-020765-10 or J-020765-12), FIP200 (J-021117-05 or J-021117-08). siGENOME RISC-Free siRNA was used as control.

### Quantitative real time PCR

Total RNA was isolated from the cells using the RNeasy kit from Qiagen and cDNA synthesis was carried out according to standard procedures. Real-time quantitative PCRs were performed using the Fast SYBR Green Master Mix (Applied Biosystems, 4385612). Forward (5′-CGG CAT TTG TTG CTC AGA ATA C-3′) and reverse (5′-AGA GAC CCG TGG ATG GAA TA -3′) primers for *MITF*, forward (5′-GCC AAC GAT CCT ATC TTC CTT C-3′) and reverse (5′-GTG CAT TGG CTT CTG GAT AAA C-3′) primers for *tyrosinase*, and forward (5′-GAC CAC TTT GTC AAG CTC ATT TC-3′) and reverse (5′-CTC TCT TCC TCT TGT GCT CTT G-3′) primers for *GAPDH* were used to generate PCR products that were detected in the 7500 FAST Real-Time PCR System (Applied Biosystems). The C_T_ values corresponding to *MITF* and *tyrosinase* mRNA were normalized to that of *GAPDH* mRNA.

### Melanin quantification

Melanin quantification was based on the protocol described by Wasmeier et al [19]. MNT-1 cells in 6-well plates were rinsed once in cold PBS, collected in 300µl of sonication buffer per well (50 mM Tris-HCl pH 7.4, 2 mM EDTA, 150 mM NaCl, 1mM DTT and protease inhibitors (Roche)) and disrupted by sonication. Lysates were centrifuged at 20,000 g for 15 min at 4°C to separate the melanin. The supernatant was taken for protein determination using Protein DC Assay (Bio-Rad), while the melanin pellets were rinsed once in ethanol/ether (1:1) and dissolved in 2 M NaOH/20% dimethylsulfoxide at 60°C. Melanin content was measured as optical density at 492nm normalized to the protein content in each sample. All experiments were repeated at least three times in duplicates and significance was determined using one-way ANOVA.

### Electron microscopy

Cells were fixed in 2.5% glutaraldehyde/4% formaldehyde in 0.1 M phosphate buffer (PB) for 1 h. The samples were postfixed in reduced osmium tetroxide, stained with tannic acid, dehydrated stepwise to 100% ethanol, and embedded in Epon. Sections (~70 nm) were cut using an Ultracut UCT ultramicrotome (Leica Microsystems), and poststained with lead citrate. Sections were viewed using a Tecnai G2 Spirit 120-kV transmission electron microscope (FEI Company) with either an Orius or an Ultrascan 1000 charge-coupled device camera (Gatan UK).

### Western Blot

Cells were lysed in ice-cold TNTE buffer (20 mM Tris, pH 7.5, 150 mM NaCl, 0.3% wt/vol Triton X-100, 5 mM EDTA) containing EDTA-free Complete Protease Inhibitor cocktail (Roche). Lysates were cleared by centrifugation and resolved on NuPAGE®Bis-Tris 4–12% gels (Invitrogen) followed by transfer onto a PVDF membrane (Millipore). Following incubation with primary and secondary antibodies the blots were either scanned with a LiCor Odyssey imager or developed by enhanced chemiluminescence (GE Healthcare). Quantification was performed with ImageJ (National Institutes of Health) and statistical significance was determined by a paired student’s t-test.

## Results

### ULK1 knockdown increases melanin content in MNT-1 cells

**Figure 1:**
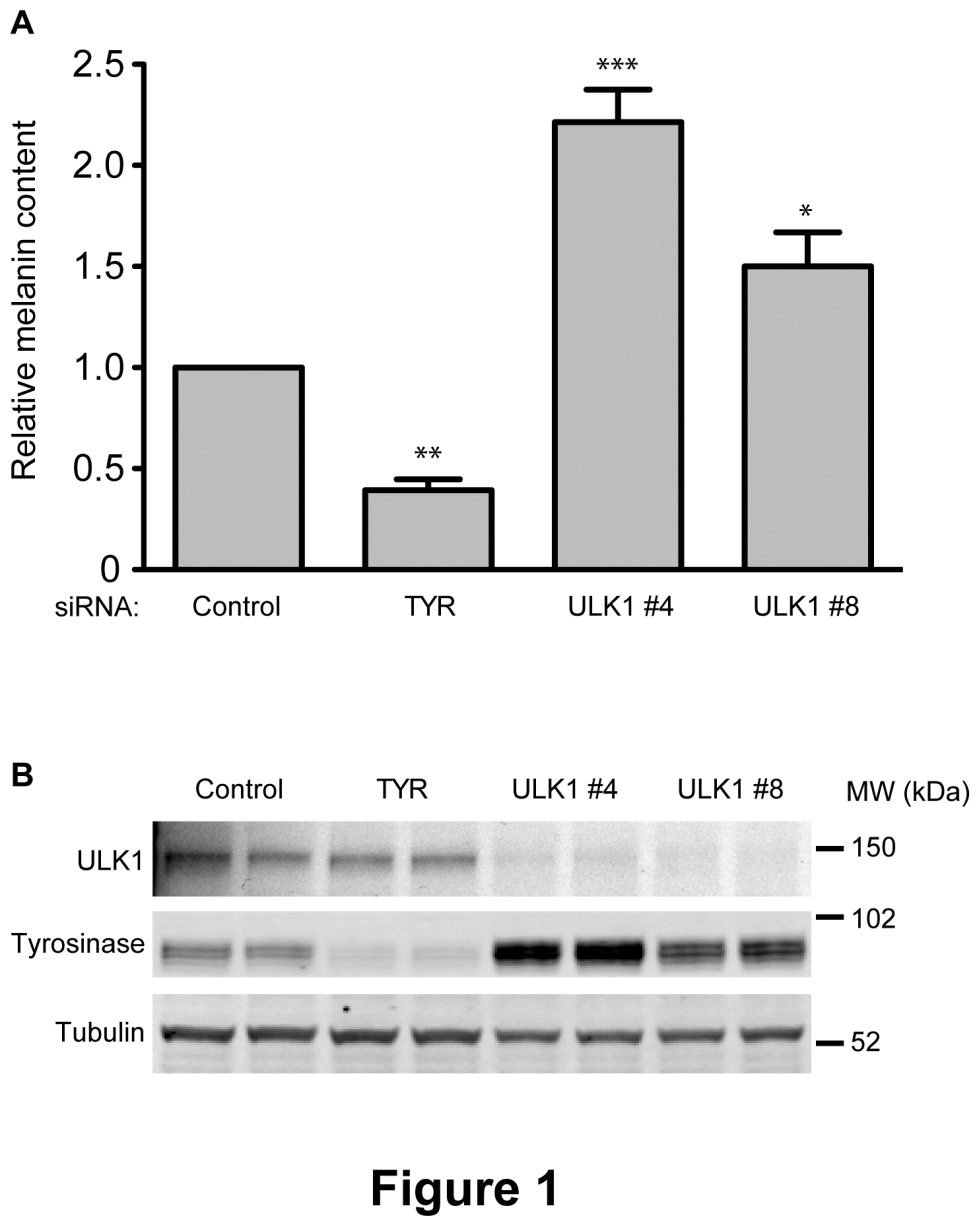
ULK1 knockdown increases melanin in MNT-1 cells. (A) MNT-1 cells were treated with a control siRNA, two ULK1-specific siRNAs and a TYR-specific siRNA pool as a positive control for changes in melanin levels. Three days after the second knockdown cells were harvested and their melanin content was analysed by measuring melanin/protein values for each sample. Significant changes relative to control were determined by a one-way ANOVA test based on three individual experiments performed in duplicates. * p<0.05; ** p<0.01; *** p<0.001; data are presented as the mean of values normalized to control within each experiment. (B) Western blot analysis of the siRNA-treated MNT-1 cells verifying the knockdowns. Samples are loaded in duplicates. Tubulin is used as a loading control.

The recent discovery of a connection between melanogenesis and the autophagy machinery by Ganesan et al [13], and the proposed model by Ho et al suggesting that WIPI1 acts as a positive regulator of transcription through mTORC1 [14], led us to investigate the role of ULK1, a mTORC1-regulated autophagy protein, in melanogenesis. In order to test this, we depleted ULK1 in MNT-1 cells and measured their melanin content by determining 492nm absorbance values and normalizing these to the sample’s protein content. The knockdown of TYR, which is the rate-limiting enzyme in melanogenesis, was included in these assays to control for melanin quantification. We found that the relative melanin content in ULK1-depleted cells compared with control cells is significantly increased (Figure 1A). This effect was verified using two individual siRNA duplexes targeting ULK1, both of which depleted the protein (Figure 1B) with duplex #4 giving a stronger effect on melanin and therefore used in follow-up assays. Knockdown of the ULK1 homologue ULK2 did not result in a significant effect on melanin levels, alone or in combination with ULK1 knockdown (data not shown). Notably, the primacy of ULK1 over ULK2 is evident also in their autophagy function in HEK293 cells, where ULK1 knockdown inhibits autophagy while ULK2 knockdown has no effect [15].

### ULK1 plays a role in melanogenesis independent of its autophagy partners and the mTORC1 pathway

**Figure 2:**
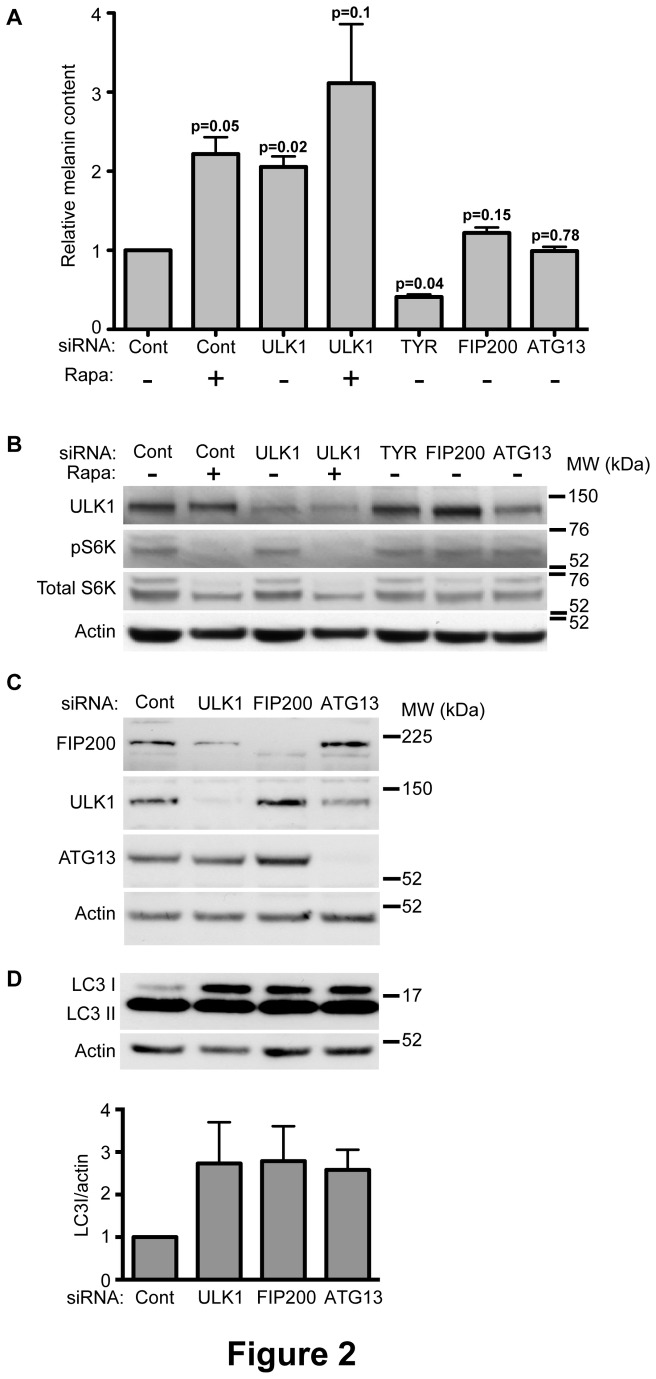
The effect of ULK1 depletion on melanin levels is independent of mTORC1 or the ULK1-ATG13-FIP200 complex. (A) Cells were treated with ULK1, FIP200, ATG13, TYR or control siRNA. Note: the data from one duplex is shown for FIP200 and Atg13, and a second siRNA duplex for each had identical effects. siRNA transfections were performed on day 1 and 3 after plating, and melanin was quantified on day 6. Control and ULK1 knockdown cells were incubated with 100nM Rapamycin (Rapa, +) or DMSO (-) starting from day 1. The data shown represents at least three individual experiments performed in duplicates, and values are normalized to control samples within each experiment. p values for the differences between the treatments and control were determined by a paired student’s t-test. (B) Cells treated as described in (A) were subjected to western blot analysis. mTORC1 activity was monitored by S6K phosphorylation, showing no significant effect after ULK1 depletion. (C) siRNA depletion of cells treated as in (A) was verified by western blot analysis. (D) Changes in basal autophagy were monitored by LC3-I and LC3-II levels, indicating an inhibitory effect for ULK1, ATG13 and FIP200 depletion in these cells. LC3-I levels were quantified by ImageJ and normalised to actin. Data shown is the mean of values normalized to actin from 4 independent experiments. Errors are SEM.

ULK1 functions in autophagy as part of a complex along with three additional autophagy proteins: FIP200, ATG13 and ATG101. To better define the context in which ULK1 regulates melanogenesis, we set out to determine the importance of the two well-characterized binding partners, FIP200 and ATG13. Depletion of FIP200 or ATG13 in MNT-1 cells using two siRNA duplexes per protein did not have a significant effect on melanin levels compared to cells transfected with control siRNA (Figure 2A), suggesting that ULK1 may act in this process through a population of the protein that is distinct from the autophagy-inducing ULK1-FIP200-ATG13 complex. As previously reported [16], siRNA depletion of Atg13 leads to decrease in ULK1 levels. However, the decrease we observed in ULK1 after Atg13 depletion did not increase melanin levels.

We looked further into the relationship between ULK1 and mTORC1, a known regulator of this complex, in the context of melanogenesis. While mTORC1 is known to negatively regulate the ULK1-FIP200-ATG13 complex, additional data also positions ULK1 upstream of mTORC1 acting in a negative feedback loop upon autophagy induction [20,21]. In light of the complex relationship between ULK1 and mTORC1, and given our finding here that ULK1 inhibits melanogenesis similarly to mTORC1, we asked whether they act in the same pathway. We hypothesized that if the effect of ULK1 knockdown occurs through the same pathway that is activated by mTORC1 inhibition, then ULK1 depletion should have no additive effect when combined with mTORC1 inhibition. We therefore measured the combined effects of ULK1 knockdown and treatment with rapamycin, an inhibitor of mTORC1, on melanin levels (Figure 2A and B). We discovered that combining the two treatments led to increased melanin levels that was greater than each of the treatments alone, suggesting that ULK1 and mTORC1 act through separate pathways to inhibit melanogenesis. Furthermore, ULK1 knockdown on its own did not have any effect on mTORC1 activity in MNT-1 cells as monitored by the phosphorylation of the mTORC1 substrate S6K1 (Figure 2B). We also probed for changes in AKT phosphorylation as a readout for the activity of mTORC2, a complex that has recently been suggested to regulate the transcription of melanogenic proteins through MITF [14]. However, we did not observe a significant effect after ULK1 knockdown on AKT phosphorylation (data not shown).

To validate the importance of the ULK complex in autophagy in MNT-1 cells, we examined the effect of ULK1, FIP200 and ATG13 depletion on basal LC3-I and II levels (Figure 2C and D). The lipidation of LC3-I to its membrane-bound form LC3-II is a key step in autophagy and is used as readout for cellular autophagy levels [22]. Indeed, knocking down each of these proteins resulted in inhibition of LC3-I to LC3-II conversion leading to an increase in LC3-I levels, confirming that ULK1, ATG13 and FIP200 are indeed active in basal autophagy in MNT-1 cells (Figure 2D).

### Melanin accumulation in ULK1 depleted cells is accompanied by increased TYR levels

**Figure 3:**
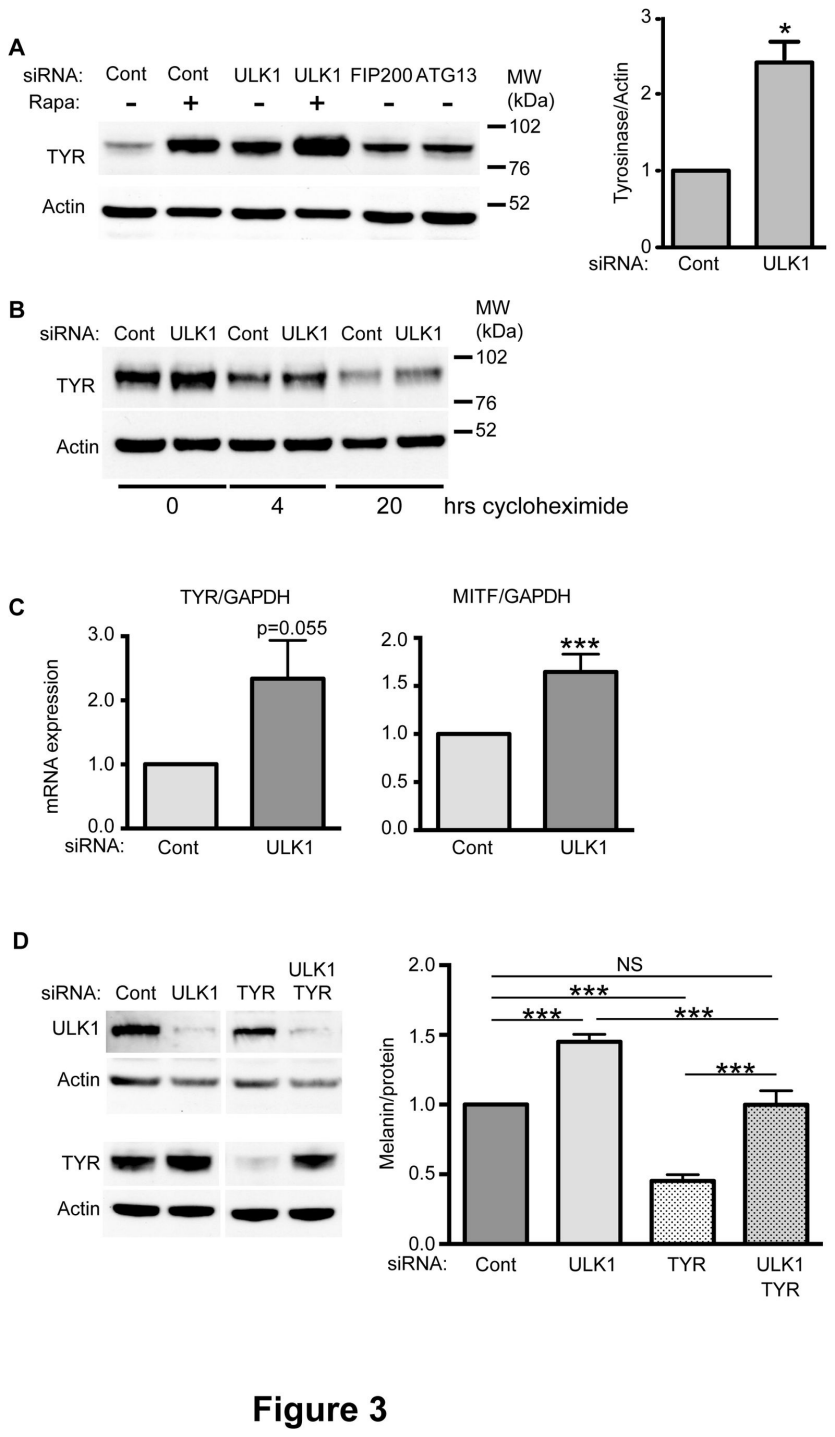
ULK1 knockdown increases TYR levels. (A) Cells were treated with ULK1, FIP200, ATG13, TYR or control siRNA and Rapamycin (Rapa, +) or DMSO (-) as indicated. Samples were subjected to Western blot analysis. TYR levels in control cells or ULK1-depleted cells were quantified using ImageJ and normalized to tubulin. Data are presented as the mean of values normalized to control within each experiment: * p<0.05; paired student t-test based on four individual experiments. (B) Cells were treated as in (A) with ULK1 or control siRNA. 4 or 20 hrs before harvesting, cells were treated with 100µg/ml cycloheximide added to growth medium. Cells were lysed and processed for western blot for TYR and actin. No difference was detected over 5 independent experiments. (C) Cells were treated as in (A) and harvested and processed for quantitative RT PCR. Data is the mean of 5 independent experiments done in triplicate; statistical significance was determined using an unpaired t-test, **, p<0.01. (D) Cells were treated as in (A) with control, ULK1, TYR or ULK1 and TYR siRNAs, and harvested for western blot for ULK1, TYR and actin or melanin quantification. Data is the mean of 5 experiments normalized to control: *** p<0.001, NS not significant, determined by a one-way ANOVA test.

TYR is the rate-limiting enzyme in melanin synthesis, and the formation of mature melanosomes is highly dependent upon the cellular abundance of this enzyme. We asked whether the changes in melanin levels in ULK1-depleted cells are accompanied by corresponding changes in TYR levels. As shown in Figures 1B and 3A, a significant increase in TYR protein levels was observed following ULK1 knockdown in MNT-1 cells compared with control cells. TYRP1, on the other hand, remained unaffected by ULK1 knockdown (data not shown). This increase correlated with the level of melanin increase observed for this treatment (Figure 1A). A similar correlation was also observed in cells where ULK1 depletion was combined with rapamycin treatment. Importantly, the knockdown of ATG13 or FIP200 did not result in a similar effect (Figure 3A), in agreement with the lack of effect on melanin levels in cells depleted from these proteins. We tested if the stability of TYR was affected by the siRNA depletion of ULK1 by performing a cycloheximide chase experiment (Figure 3B). After control or siRNA depletion of ULK1 we observed no difference in the stability of TYR at 4 or 20 hrs of incubation.

Although there is no published data implicating ULK1 either positively or negatively regulating transcriptional responses, we checked if ULK1 depletion affected the transcription of tyrosinase. We found that ULK1 depletion increased mRNA levels for both TYR and the upstream transcription factor, Microphthalmia-associated Transcription Factor (MITF) (Figure 3C). In accordance with this, we tested the effect of a double knockdown of ULK1 and tyrosinase (Figure 3D). As expected melanin levels increased or decreased with depletion of ULK1 and TYR, respectively. After simultaneous depletion of ULK1 and TYR we found melanin levels were not significantly different from control levels. As TYR protein levels increased to control levels in the double knockdown, we believe the restoration of melanin levels reflects an increase in transcription of TYR driven by depletion of ULK1, which compensates for the decrease caused by the siRNA depletion. In summary, the increase in melanin levels after depletion of ULK1 is likely due to the effects of ULK1 on the transcription of MITF and TYR, and the subsequent increased production of TYR.

### ULK1-depleted cells contain larger melanosomes

**Figure 4:**
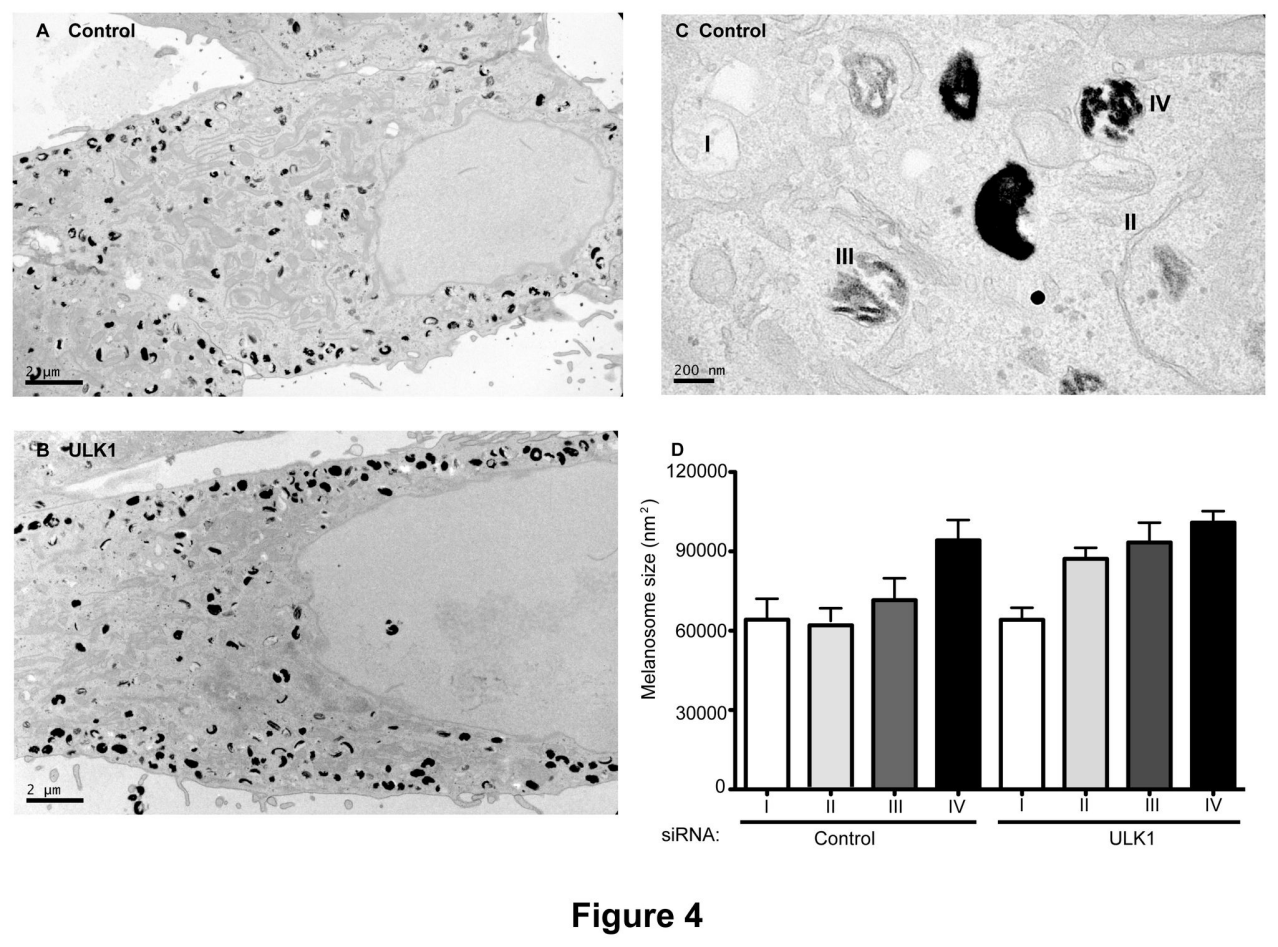
EM analysis of ULK1 knocked-down cells reveals larger melanosomes. Cells were treated with (A and C) control or (B) ULK1 siRNA and prepared for EM analysis. For quantification (D) of melanosome number and area, cells were blindly chosen. A series of images were taken within each cell to cover a similar area between all cells at a 49,000X magnification. Over 80 images from total of 15 cells per condition were collected. The number of melanosomes at different stages (shown in control cells C) was manually scored within each cell. (D) Average melanosome size of the melanosomes in each stage in control and ULK1 siRNA treated cells.

To gain further insight into the effect of ULK1 depletion on melanogenesis we characterised ULK1 knockdown cells by electron microscopy, as the increase in melanin could be due to either an increased number of melanosomes or an increase in the size of the melanosome. Morphologically, the melanosomes in ULK1 knocked-down cells did not look any different than the ones in control cells (Figure 4A and B). We also could not detect any increase in the number of melanosomes in ULK1-depleted cells, although we cannot rule out the possibility that the numbers might be higher as there are limitations in the EM analysis, in particular with sampling. However, analysis of the size of melanosomes at different stages of development (Figure 4C) revealed that ULK1-depleted cells tend to have larger stage II and III melanosomes (Figure 4D). Therefore although we could not detect a significant change in the number or morphology of the melanosomes, the increased size of stage II, III and to lesser extent stage IV melanosomes may explain, at least in part, the overall increase in melanin content of the cells.

## Discussion

Melanin synthesis requires the sequential delivery of specific proteins to the forming melanosome through transport mechanisms that are not yet fully understood. This delivery relies on membrane trafficking machinery that may overlap with other cellular pathways, in conjunction with melanocyte-specific proteins. The recent discovery of a connection between melanogenesis and the autophagy machinery and the proposed model placing WIPI1 as a positive regulator of transcription through mTORC1 [14], led us to investigate the role of ULK1, an mTORC1-regulated autophagy protein, in melanogenesis. The data presented here supports an inhibitory function for ULK1 in the regulation of melanin production in melanocytes. Importantly, the depletion of FIP200 and ATG13, members of the ULK1 complex that is activated upon autophagy induction, did not have an effect on melanin levels in MNT-1 cells, suggesting that ULK1 is involved in melanogenesis independently of its role in autophagy. This possibility is further supported by the observation that while ULK1 knockdown increases TYR, the knockdown of ATG13 and FIP200 does not result in a similar effect (data not shown). However, as seen in Figure 2C, Atg13 knockdown decreases the level of ULK1. We speculate that either this reduction in ULK1 levels is not sufficient to cause the increase in transcription of MITF or TYR, or that the loss of ULK1 caused by depletion of Atg13 occurs gradually and has less impact on melanin levels than acute depletion of ULK1 using the specific siRNA. Either way there must be a critical threshold of ULK1 levels to cause a change in transcription. In addition, the effects of ULK1 depletion on melanin and TYR levels were additive with rapamycin treatment, and were not accompanied by a change in TOR signalling, suggesting that ULK1 exerts its effects through distinct pathways of those induced by mTORC1.

The increase in TYR levels in ULK1-depleted cells may provide a clue to the mechanism by which ULK1 regulates melanin levels. TYR is the rate-limiting enzyme in melanin synthesis, and as such an increase in TYR levels can be sufficient to induce an increase in total cell melanin [23,24]. The main regulator of TYR expression is MITF, which is the key transcription factor for the induction of melanogenesis following a variety of stimuli. We found an increase in the transcription of both TYR and MITF in ULK1-depleted cells suggesting this may be the mechanism by which ULK1 increases melanin. In line with this a depletion of both ULK1 and TYR together restored melanin levels the control levels suggesting a direct effect of ULK1 on TYR. In addition, the stability of the TYR was not affected by ULK1 depletion making it likely the effects were primarily through transcriptional control. Our results showing an increase in the transcription of MITF after depletion of ULK1 are in fact in agreement with previously published results: Ho et al observed a 1.4 fold-increase in MITF mRNA levels after depletion of ULK1 using a pool of three siRNAs [14].

Our data reveals a more complex relationship between melanogenesis and autophagy than anticipated. Ganesan et al have shown that melanogenesis may require autophagy machinery components for its progression in MNT-1 cells, and WIPI1 was later suggested to play a positive role in this process [13]. Indeed, the effect of WIPI1 depletion on melanin levels was also robust in our hands (unpublished observations), but the effect of ULK1 depletion revealed an inhibitory role for this key autophagy protein during melanogenesis. However, the effect of depletion of autophagy genes on production of melanin in vivo, and in particular on coat color, has so far not been reported, except for Beclin1-/+ mice which have a lighter coat color [13]. We have not seen any alteration of coat color in our ULK1-/- bred on a BL6 background (unpublished observations). We presume that if there was an increase in the melanin level in the ULK1-/- mice this was not sufficient to cause a gross alteration of the coat color. It may also be that ULK1 and 2 play a redundant role in vivo and that depletion of both genes would be required for alterations in coat color. This remains to be tested in a conditional model as the ULK1-/-ULK2-/- mice do not survive [25].

It is likely that at steady-state a balance exists between autophagy-related production and degradation of melanosomes, in which proteins of the autophagy machinery with a role in both processes are recruited to either one depending on cellular requirements. Murase et al have recently demonstrated that melanosomes are degraded by autophagosomes under basal conditions in keratinocytes [26]. As autophagy is a conserved process found in virtually all cell types in the body, it is conceivable that autophagy will also function as a catabolic pathway in melanocytes. This scenario raises several questions regarding the way melanocytes may deploy the same machinery for the two opposite outcomes, i.e. the energy-demanding production of melanosomes versus their degradation, and to whether this balance is determined by metabolic considerations, melanin levels, or both. Such questions will inevitably become clearer as we gain better understanding of the molecular mechanisms behind the regulation of melanogenesis by autophagy proteins.
